# Being social, being socially constructed, and being fundamental relative to social reality

**DOI:** 10.1007/s11098-025-02446-1

**Published:** 2025-11-28

**Authors:** Emilie Pagano

**Affiliations:** Binghamton University, Binghamton, USA

**Keywords:** Being social, Being socially constructed, Social construction, Social reality, Relative fundamentality

## Abstract

Although the properties of being social and of being socially constructed are indispensable to our understanding of social reality, social metaphysicians are unclear about how they’re related. In this paper, I argue that whereas everything that’s socially constructed is also social, not everything that’s social is also socially constructed. In particular, I argue that something is what I call “fundamental relative to social reality,” something that’s social but not also socially constructed. I sketch an account of fundamentality relative to social reality, and argue that it both clarifies how the properties of being social and of being socially constructed are related, and captures their significance for our understanding of social reality per se.

## Introduction

The properties of being social and of being socially constructed are indispensable to our understanding of “social reality,” that bit of “big-R” reality that contains things like aristocrats, armories, and Art Basal, bologna, beauticians, and Boston, and coroners, colleges, and carnivals. For it’s important we recognize that in addition to containing goings-on^1^ like them – namely, *social* ones – social reality has structure. In particular, it’s important we recognize that some of the social goings-on it contains depend on others; in other words, that some of them are *socially constructed*.

For instance, we can assume that social reality in some sense contains the fact that the Department of Philosophy is on strike – a social thing – and that this fact depends on the fact that the majority of its members are engaged in a coordinated refusal to work – another social thing. Thus, we can assume that the fact that the Department of Philosophy is on strike is social and also socially constructed. But we should ask: Is *everything* that’s socially constructed also social? And: Is *everything* that’s social also socially constructed? Interestingly, social metaphysicians haven’t said.^2^

To some extent, that’s unsurprising. Explicit interest in the metaphysics of social construction (in the relevant sense) is relatively recent.^3^ For instance, although John Searle’s *The Construction of Social Reality* (1995) might be read as an account of social construction, his use of the term is more or less confined to its title. But even supposing his account of the construction of social reality should be read as an account of social construction (in the relevant sense), it’s unclear how he’d answer our questions. In particular, it’s unclear whether what’s distinctive in it – specifically, whether Searlesian collective intentions – are intended to be social^4^ and/or socially constructed.

Understandably, the same unclarity appears in the work of Searle’s commentators. For instance, both Amie Thomasson (2003) and Muhammed Ali Khalidi (2013) emphasize that Searle’s account applies exclusively to what Thomasson calls social “products,” and what Khalidi calls “Type 2” and “Type 3” social kinds, respectively, each of which depends on Searlesian collective intentions. Each argues that Searle’s account thereby ignores particular kinds of social goings-on – what Thomasson calls social “byproducts,” and what Khalidi calls “Type 1” social kinds, respectively – each of which can exist whether or not we intend them to.^5^ But, again, it’s unclear whether social byproducts and Type 1 social kinds are themselves socially constructed in the relevant sense. For, again, supposing we should read both Thomasson’s and Khalidi’s as accounts of social construction, it’s unclear how they’d answer our questions. For all they say, both the product/byproduct and Type distinctions crosscut the social/socially constructed distinction.

Nonetheless, it *is* to some extent surprising that social metaphysicians haven’t said whether everything that’s socially constructed is also social, or whether everything that’s social is also socially constructed. In particular, it’s surprising that social metaphysicians with *explicit* interest in the metaphysics of social construction (in the relevant sense) haven’t said. Sally Haslanger is an exemplar. For she characterizes the project of identifying what’s socially constructed (i.e., the “social constructionist project” (Haslanger, 2012: 83)) as aimed at revealing what’s social rather than natural. In particular, she claims that although it isn’t transparent, “our efforts to classify [e.g., race and gender] as ‘natural’…have failed [because] they ignore the force of social construction (Haslanger, 2012: 83).”^6^ It’s therefore tempting to read Haslanger as seeing the properties of being social and of being socially constructed as equivalent: race and gender are social rather than natural *because they’re socially constructed*, and they’re socially constructed *because they’re social rather than natural*.^7^ But whether that’s what she intends is unclear.

Of course, Haslanger might insist that although everything that’s *non-transparently* social is also socially constructed, it’s not the case that everything that’s *transparently* social is. Maybe race and gender are socially constructed because we don’t *see* that they’re social, and maybe they’ll cease to be socially constructed when we do.^8^ That’s an intriguing possibility. But the point now isn’t that we’re short on intriguing possibilities, especially if we countenance a distinction between the properties of being social and of being socially constructed. The point is precisely that we haven’t countenanced a distinction, and so that we haven’t been intrigued by the possibilities that distinction reveals.

In light of all of this unclarity, I’ll consider whether we ought to accept.

**SC to S**: If x is socially constructed, x is social

and/or.

**S to SC**: If x is social, x is socially constructed

And I’ll argue that whereas everything that’s socially constructed is also social – that is, that we ought to accept **SC to S** – not everything that’s social is also socially constructed – that is, that we ought not to accept **S to SC**.

Here’s how it’ll go. In **§II**, I identify target phenomena for accounts of both the property of being social and the property of being socially constructed. In **§III**, I articulate a model of social reality, and, in **§IV**, I argue that it implies that **SC to S** is true, and that **S to SC** is false. In particular, I argue that **S to SC** commits us to a view we shouldn’t accept; namely, that social reality consists of an infinite series of social constructions. In response, I argue that something (that’s social but not also socially constructed) has to be what I call “fundamental relative to social reality”. In **§V**, I propose an account of fundamentality relative to social reality using Searle’s account as an illustration. I conclude in **§VI**. Ultimately, I aim to have shown that by distinguishing the property of being social from the property of being socially constructed, we’ll have availed ourselves of a distinction that’s of use to social metaphysicians quite generally.

A note on terminology. In general, I’ll speak of social and socially constructed *facts* rather than of social and socially constructed *things*, and I’ll frequently use “[x]” to express “The fact that x”. This is merely for convenience. Ultimately, I’m happy to admit that things are socially constructed in addition to – or even rather than – facts. But insofar as we can refer to an object indirectly by way of the fact that it exists, I’ll hold on to that convenience.

## Target phenomena

In this section, I identify target phenomena for accounts of both the property of being social and of the property of being socially constructed. Of course, to identify a target phenomenon for an account of P isn’t to account for P, but to circumscribe the phenomenon of which one’s account is an account. My aim therefore isn’t to account for either property, but to ensure that the model of social reality I articulate in **§III** is appropriately neutral, that different accounts of both the property of being social and of the property of being socially constructed have a right to it.

### Being social

Ordinarily, we identify target phenomena by way of the roles they’re intended to play in our theorizing. For instance, we say that a particular set of individuals is a *group* (rather than a mere set of individuals) when we want to explain the fact that it can survive changes in membership. Similarly, we say that gender is “the social meaning of sex” (rather than that it *is* sex) when we want to explain the fact that the relevant sets of individuals are *treated* in predictable ways. And so on. But it’s unclear precisely which role the property of being social is intended to play in our theorizing. So, the question is: in theorizing about the property of being social, what do we mean to be theorizing about?

That it’s unclear likely explains social metaphysicians pessimism about the possibility of *defining* the property of being social (Epstein, 2015; Haslanger, 2012; Ritchie, 2020). As Katherine Ritchie suggests,

[g]iving a non-circular definition of what it takes for something to be social is difficult, if not impossible. While being social is obviously connected to society…without a definition of ‘society’ that do[es] not appeal to ‘social’ we are no closer to a definition. (Ritchie, 2020: 404)

Clearly, if to be social is to be connected to society, we’ve defined being social in terms of a social thing. But if we’ve defined being social in terms of a social thing, we haven’t accounted for it. And although being connected to society doesn’t exhaust the possible definitions of being social, it’s difficult to see what could do it. Surely, being social has something to do with a kind of *interaction*, but in a sense of “interaction” that’s distinguished from e.g., biological, chemical, and physical kinds of interaction; for instance, between the kinds of interaction that characterize evolution, bonding, and collision, respectively. But that’s the problem. *In that sense*, the kind of interaction with which the property of being social has something to do is bound to be as social as evolution, bonding, and collision are biological, chemical, and physical, respectively. If so, we’re back to defining the property of being social in terms of a social thing.

As a result, it’s common to identify the social things there are contrastively. In particular, it’s common to “mark” the social things there are by identifying a set of properties they have that a particular set of *non*-social things don’t. Again, something is distinctive about the kind of interaction associated with being social, and its distinctiveness emerges in contrast to “natural”^9^ (e.g., biological, chemical, and physical) kinds of interaction. Social things like syndicates, the Salsa, and strikes are the kind of thing that in some sense concern “us”. (I’ll say more about who “us” refers to in **§IV.i.**.) They’re relevantly *unlike* paradigmatically natural things, things like e.g., snails, sodium, and space that *don’t* concern us in the relevant sense. Thus, we can identify social things by pointing to pairwise paradigms: “*These* belong to the social domain” and “*Those* belong to the natural domain,” we say, and then kick away the ladder.

In this spirit, propose we accept.

**Social**: if x is social, x doesn’t belong to the natural (e.g., the biological or chemical or physical, etc.) domain

However, it’s important to note that **Social** doesn't yet provide a target phenomenon for accounts of the property of being social. First, we need a way of fixing reference to the relevant domains. For we can’t think of the social things there are simply as the *non-natural* things there are, or of the natural things there are as simply the *non-social* things there are.^10^ If an exclusive contrast like ours succeeds in picking something out, it certainly isn’t guaranteed to pick out the target phenomenon we intend our pointing to pick out. Thus, we need a sense of the kinds of properties to which the “relevantly like” in “relevantly like syndicates, the Salsa, and strikes” are attuned, since these are precisely the kinds of properties to which the “relevantly *un*like” in “relevantly unlike snails, sodium, and space” are attuned, too. Of course, we can disagree about which properties to prefer (and, of course, we have).^11^,^12^ But for the sake of definiteness, I’ll suggest one. Roughly: whereas social things are the kind of thing whose existence requires *us* – in the sense that they depend on things we do (in some appropriately wide sense of “do”) – natural things are the kind of thing whose existence doesn’t.^13^

Consider syndicates, the Salsa, and strikes, on the one hand, and snails, sodium, and space, on the other hand. Syndicates, the Salsa, and strikes depend on things we do (in some appropriately wide sense of “do”). We can create syndicates with handshakes, destroy the Salsa with censorship, and affect the outcomes of strikes by agreeing to terms. But snails, sodium, and space don’t depend on things we do in this way. Try as we might, we can’t create snails by anything like shaking hands, we can’t destroy sodium by anything like censoring it, and we can’t affect space by anything like agreeing to terms. Of course, what we do can *cause* natural things to exist, to cease to exist, and to change in various ways. It’s uncontroversial that we can create ecosystems and thereby cause snails to proliferate, that we can mine sodium to such an extent that there comes to be no sodium, and maybe even that we can invent technology that warps space in radical ways. However, we don’t merely *cause* our social paradigms to exist, to cease to exist, or to change. What we do is in some sense part of *what it is* to be a syndicate, the Salsa, and a strike. Syndicates depend on things we do in the sense that the fact that we’re a syndicate, say, is grounded in the fact that we formally agree to pool our resources; for instance, by way of shaking hands. Likewise, the fact that the relevant dance practice (i.e., the Salsa) exists is grounded in the fact that we move in quite particular ways on quite particular occasions, and this might come to be prohibited; for instance, as a result of censorship. Lastly, the fact that we’re on strike is grounded in our being engaged in a coordinated refusal to work, and we might change our tactics; for instance, should we agree to certain terms. But the point is: natural things aren’t like *that*.

Clearly, that’s a sketch; more needs to be said to defend the relevant “mark”. As clearly, **Social** isn’t *committed* to the view that depending on things we do is what distinguishes the social from the natural. But when we go looking for an account of the property of being social, **Social** tells us where to look. It tells us that whatever it is to be social, and whatever it is to be natural, the social and the natural things belong to exclusive domains. And insofar as we can use something’s depending on things we do in the relevant way to fix reference to these domains, we can say: if x is social, x isn’t natural, because if x is social, x depends on things we do in a way that natural things can’t. Assuming it’s legitimate to speak of exclusive social and natural domains, **Social** allows us to ask the questions with which we began without presupposing an account of the property of being social.

### Being socially constructed

Luckily, social metaphysicians are clear about which role the property of being socially constructed is intended to play. Some things depend on social goings-on, and that can be quite surprising. For instance, many find it surprising to learn that e.g., race and gender are relevantly like syndicates, the Salsa, and strikes in this way. Social metaphysicians they therefore agree to use “social construction” to refer to the way in which something might (and might not!) depend on social goings-on (cf. Haslanger, 2012; Epstein 2016, 2024; Mallon, 2016, 2024; Ásta 2018; Schaffer, 2017, 2019; Griffith, 2017, 2018; Passinsky 2020, *Forthcoming*; Pagano *Forthcoming*).

In particular, they agree that when we claim that one thing constructs a second thing, although we might disagree about both the kinds of things that construct (i.e., general kinds of things like facts or concepts, on the one hand, and particular things like syndicates, the Salsa, or strikes, on the other hand) and about the kinds of things that are constructed (same), construction is a kind of dependence. Thus, although different views about what constructs, what’s constructed, and the kind of dependence in question will result in different accounts of social construction, these accounts have a basic view in common: *if x is socially constructed, x depends on social goings-on*.

Of course, “dependence” isn’t univocal. There are varieties. And in the literature, it’s standard to distinguish two basic varieties of social construction that correspond to two basic varieties of dependence: causal and non-causal (or: “constitutive”)^14^ social construction.

On the one hand, to be causally socially constructed is at least to partly causally depend on social goings-on. For instance, the Department of Philosophy’s strike might be socially constructed in the sense that their employers’ mistreatment might cause the majority of its members to coordinate in the relevant ways; say, to pamphlet, picket, and/or protest. In that case, their strike is causally socially constructed by their employers’ mistreatment. On the other hand, to be non-causally socially constructed is at least to partly non-causally depend on social goings-on. For instance, the Department of Philosophy’s strike might be socially constructed in the sense that strikes *are* coordinated refusals to work. In other words, the fact that the Department of Philosophy is on strike might be socially constructed in the sense that it holds *in virtue of* the fact that they’re engaged in a coordinated refusal to work, *that* they’re pamphleting, picketing, and/or protesting. And, the fact that the majority of its members are engaged in a coordinated refusal to work doesn’t *cause* them to strike. Rather, the fact that the Department of Philosophy is on strike is non-causally socially constructed by it.^15^

Although causal social construction is of interest – especially in explaining how our lives are *shaped* by social goings-on, and especially in ways that aren’t transparent (Haslanger, 2012: Ch. 2) – non-causal social construction is of special interest. For as claims about causal social construction, **S to SC** and **SC to S** are uninterestingly false.

Assuming **Social**, it’s uncontroversial that some natural things causally depend on social goings-on. Again, we can create ecosystems and thereby cause snails to proliferate, we can mine sodium to such an extent that there comes to be no sodium, and maybe we can invent technology that warps space in radical ways. In each case, something natural causally depends on something we do. It’s similarly uncontroversial that some social goings-on causally depend on natural goings-on. For instance, the fact that snails have proliferated can cause us to campaign for reform, that there’s no sodium can cause McDonald’s to go out of business, and that space is e.g., six-dimensional can cause us to have cooler parties. Again, in each case something social causally depends on natural goings-on. Thus, not everything that’s causally socially constructed is also social, and not everything that’s social is also causally socially constructed. Causal interpretations of **S to SC** and **SC to S** are therefore false, but uninterestingly so.

The questions of interest to us concern whether everything that’s social also *non*-causally depends on social goings-on, and whether everything that *non*-causally depends on social goings-on is also social. As a result, I propose we accept.

**Social Construction**: if x is socially constructed, x non-causally depends (henceforth: depends) on social goings-on

Unlike **Social**, **Social Construction** doesn’t need to be supplemented to provide a target phenomenon for accounts of the property of being socially constructed. Social metaphysicians have offered accounts of social construction that *self-consciously* conform to it.,^16^,^17^ As with **Social**, **Social Construction** tells us where to look when we go looking for an account of the property of being socially constructed. And as with **Social**, it allows us to ask the questions with which we began without presupposing an account of the property of being socially constructed.

### SC to S & S to SC

Finally, **Social** and **Social Construction** allow us to refine our questions. When we ask whether everything that’s socially constructed is also social (**SC to S**), we’re asking whether everything that non-causally depends on social goings-on belongs to the social rather than the natural domain. Again, we *aren’t* asking whether everything that *causally* depends on social goings-on belongs to the social rather than the natural domain. We agree that it doesn’t.

Likewise, when we ask whether everything that’s social is also socially constructed (**S to SC**), we’re asking whether everything that belongs to the social rather than the natural domain non-causally depends on social goings-on. We aren’t asking whether everything that belongs to the social rather than the natural domain *causally* depends on social goings-on. Again, we agree that it doesn’t.

Importantly, because we aren’t interested in accounts of either the property of being social or of being socially constructed, but in how our target phenomena are related, we need an independent basis for answering our questions. (*If* we had accounts of them, we’d have answers to our questions already!) To that end, I’ll articulate what I take to be an attractive model of social reality that’s neutral with respect to accounts of both the property of being social and of being socially constructed. With this model, we can use **Social** and **Social Construction** as guides to answering our questions. In particular, we can ask *what social reality must be like* in order for **SC to S** and **S to SC** to be true. The model therefore provides the independent basis we need to evaluate them.

## Social reality

Consider Fig. 1.

**Fig. 1:**
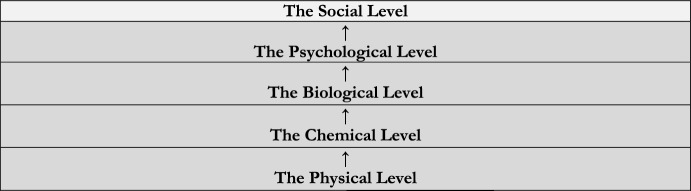
General model of reality

Figure 1 is a version of the general model of reality first articulated by Paul Oppenheim and Hilary Putnam (1958) in representing reality as consisting of *a hierarchy of levels*.,^18^,^19^

On the one hand, Fig. 1 represents reality as consisting of *kinds of things* – here the social, psychological, biological, chemical, and physical things, respectively – each of which comprises a *level*. Again, the social things are things relevantly like strikes, and the psychological things are things relevantly like schizophrenia, the biological things relevantly like snails, the chemical things relevantly like sodium, and the physical things relevantly like space. Figure 1 therefore implies that everything that’s social belongs to reality’s social level, and that everything that belongs to reality’s social level is social. Indeed, Fig. 1 implies that, for any level, L, L is individuated by a kind, K, such that, for all x, x belongs to L if and only if x is K. Thus, everything that’s natural (e.g., psychological or biological or chemical or physical) belongs to one of reality’s “natural levels” (i.e., its psychological, biological, chemical, or physical level). Levels are therefore exclusive.

On the other hand, Fig. 1 represents reality’s levels as structured in a particular way. Where ‘↑’ represents dependence, it implies that reality’s social level directly depends on its psychological level, but not conversely, which directly depends on its biological level, but not conversely, which directly depends on its chemical level, but not conversely, which directly depends on its physical level, but not conversely. Figure 1 therefore represents reality’s levels as forming a *hierarchy* that’s such that when one level either directly or indirectly depends on a second level, the first is “above” the second.^20^ Exclusive levels are therefore ordered.

That said, the general model of reality (of which Fig. 1 is a version) is quite neutral. First, it’s neutral with respect to which levels reality contains. I’ve represented it as containing exactly five: the social, the psychological, the biological, the chemical, and the physical. But we can disagree about that. For instance, it’s possible that I’ve included a level that others wouldn’t include – for instance, the psychological level (as Oppenheim and Putnam wouldn’t) – and/or that I’ve excluded a level that others would include – for instance, a moral level. Nonetheless, the general model is compatible with different accounts of reality’s basic ontology. Because it represents reality’s levels as exhaustive, one can add and subtract levels as one sees fit.

Second, the general model is neutral with respect to the kinds of things that belong to each of reality’s levels. For instance, because it’s intended to capture reality’s mereological structure, Oppenheim & Putnam’s version of the model consists of levels of particulars. However, I use “thing” broadly to include facts such that Fig. 1 captures a more general kind of structure (i.e., that consists of more than the relation between things and their parts). But virtually any kind of thing that exhibits the relevant kind of ordering (i.e., where one thing’s directly or indirectly depending on a second thing implies that the first is “above” the second) can be added, and the model adapted to accommodate it.

Relatedly, the general model is neutral with respect to the kind of dependence ‘↑’ represents. It’s possible there are many kinds of dependence involved, and each might have distinctive logical properties. Again, Oppenheim & Putman’s model is intended to capture reality’s mereological structure such that ‘↑’ represents the dependence exhibited by things and their parts. But as before, we can disagree about that.^21^

Lastly, the general model is neutral with respect to whether reality has a foundation, a final level on which everything ultimately depends. I’ve represented it as if it does, and I’ve represented its physical level as its foundation. But, again, we can disagree about that.

On the one hand, reality might not have a foundation. Reality might be “abyssal” in the sense that it “contain[s] only material wholes the existence and identity of which depends on their material part[s] (Raven, 2016: 610).” If it is, for any material whole, W, the fact that W exists depends on infinitely many further facts. Nonetheless, the view that reality is abyssal is compatible with – in fact, it presupposes – the view that reality forms the relevant kind of hierarchy. Abysses “decompose” into further and further parts such that abyssal worlds are hierarchically structured in precisely the sense in question. Whether reality has a foundation is therefore a matter of detail.

Supposing, on the other hand, that reality *does* have a foundation, we can disagree about which level/s comprise/s it. It’s quite common among those who accept the general model of reality to maintain that its physical level comprises reality’s foundation. But one might accept that some mental goings-on, that some moral goings-on, even that some social goings-on are foundational. As a result, although Fig. 1 is opinionated with respect to reality’s foundation – and, so, its ordering – the general model of reality isn’t.

In light of its flexibility, I propose we replace Fig. 1 with a version of the general model that more obviously serves our purposes. As I’ve suggested, reality consists of a social level and several natural levels: here a psychological, a biological, a chemical, and a physical level. Per **Social**, reality’s social level consists of paradigmatically social things, and its natural levels consist of paradigmatically natural things such that reality’s social and natural levels are exclusive. But reality’s levels aren’t mere heaps of things. Per **Social Construction**, reality’s social level doesn’t consist of a mere set of facts (e.g., the fact that the Department of Philosophy is on strike, and the fact that the majority of its members are engaged in a coordinated refusal to work), but of some social facts depending on others. And *mutatis mutandis* for reality’s natural levels. As a result, we replace Fig. 1 with a version of the general model that reflect this.

I propose Fig. 2.

**Fig. 2:**
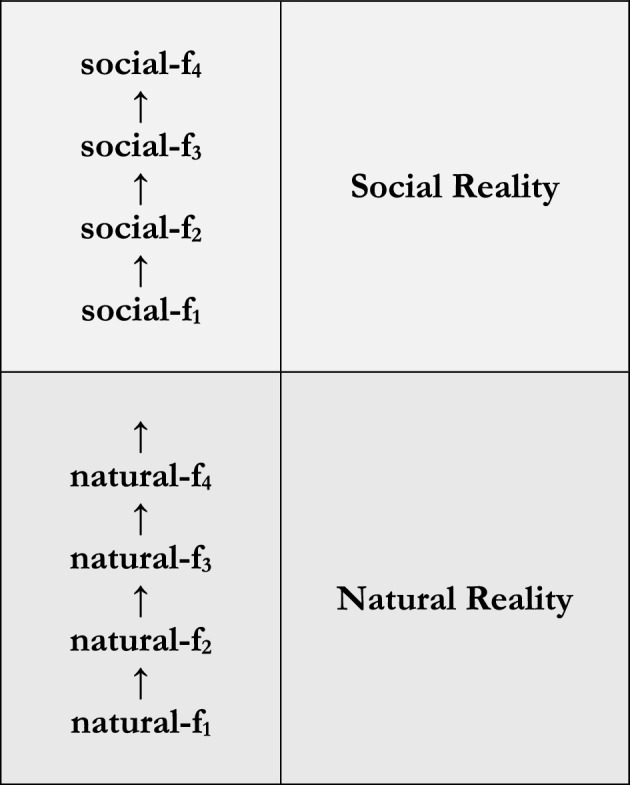
Social reality and natural reality

On the one hand, Fig. 2 conceives of reality’s natural level*s* as a single super*level* consisting of the natural levels represented in Fig. 1. Still, in conceiving of reality’s natural level*s* as a single superlevel, Fig. 2 doesn’t eliminate the former. Rather, it’s neutral both with respect to how natural reality is structured (e.g., with respect to the relations among e.g., the psychological, biological, chemical, and physical), and thereby with respect to *how* social reality depends on natural reality. For instance, it’s widely accepted that social goings-on directly depend on *both* psychological and “material” goings-on; that is, on features of natural reality out of which we create social goings-on (Haslanger, 2012; Epstein 2016). But insofar as psychological and material goings-on belong to *some* natural level, Fig. 2 can accommodate this. All it tells us is that there’s a *real* distinction between the social and the natural, and that the social depends on the natural, but not conversely.^22^

On the other hand, Fig. 2 represents reality’s levels as hierarchically related in *two* ways. First, represents reality’s levels as having internal structure. The facts that belong to each of reality’s levels are themselves hierarchically related: social-f_4_ directly depends on social-f_3_, but not conversely, which directly depends on social-f_2_, but not conversely, which directly depends on social-f_1_, but not conversely.^23^ Second, reality’s levels have external structure. As before, reality’s levels are themselves hierarchically related: social reality directly depends on natural reality, but not conversely. But now we see *why* social reality directly depends on natural reality. We see that social reality directly depends on natural reality *because* a particular social fact – namely, social-fact_1_ – directly depends on a particular natural fact – namely, natural-fact_4_, but not conversely. These facts therefore comprise a kind of nexus, a point at which reality’s levels are held together.^24^

And it’s because of each of these features that Fig. 2 substantiates our target phenomena. Exclusivity and exhaustivity are general features of the general model of reality as represented in Fig. 1. Thus, Fig. 2 supports a meaningful contrast between the social and the natural (per **Social**) given it’s an appropriately supplemented version of that model. But now Fig. 2 understands exclusivity and exhaustivity in terms of reality’s levels consisting of exclusive and exhaustive domains of *facts* that are externally related by way of social-fact_1_ and natural-fact_4_. And because social-f_4_ directly depends on social-f_3_, but not conversely, which directly depends on social-f_2_, but not conversely, which directly depends on social-f_1_, but not conversely, Fig. 2 captures facts about social construction; in depending on social goings-on, social-facts_2-4_ are socially constructed (per **Social Construction**). Thus, Fig. 2 integrates both **Social** and **Social Construction** into a general model of reality. And that’s attractive.

Nonetheless, Fig. 2 deserves to be controversial, and its controversies are many, varied, and well-documented.^25^Arguably, exclusivity is its most controversial feature.^26^ For, as with Fig. 1, Fig. 2 represents reality’s levels as exclusive in the sense that nothing is both social and natural. But it’s clear that reality in some sense contains “mixed” facts, facts that have both social and natural components. A simple example: [The Department of Philosophy is located at 37°14′0″N 115°48′30″W] has both the Department of Philosophy – a social thing – and the location referred to by “37°14′0″N 115°48′30″W” – a natural thing – as components. Figure 2 implies that this fact belongs to exactly one of reality’s levels, but it’s unclear which. For if it belongs to social reality, the natural in some sense permeates social reality; in particular, because it in some sense contains something natural (i.e., the relevant location). Likewise, if this fact belongs to natural reality, the social in the same sense permeates natural reality; again, because it in some sense contains something social (i.e., the Department of Philosophy). And likewise if it belongs to both. But in either case, we *can’t* accept that reality’s levels are non-overlapping contra Fig. 2.

Clearly, this kind of concern assumes that a fact is the kind of fact it is (e.g., a social or a natural fact) because of the kinds of things it contains. In particular, it assumes that a fact belongs to whatever level of reality its components belongs to. (Mixed facts are worrying to the extent that their components belong to different levels of reality.) But as we can disagree about which levels reality contains, which kinds of things belong to them, which kind/s of dependence holds them together, and whether they all ultimately depend on a foundation, we can disagree about that. In other words, Fig. 2 doesn’t commit us to a particular criterion for what makes a fact the kind of fact it is.

In fact, Fig. 2 is compatible with a host of criteria. For instance:

1. whether a fact is a fact of a particular kind depends on the *vocabulary* required to state it^27^
2. whether a fact is a fact of a particular kind depends on the status of its least fundamental component^28^
3. whether a fact is a fact of a particular kind depends on of what it’s directly *about*

Each of these has different implications for whether mixed facts of the relevant sort are social, natural, or both. This in turn has implications for whether exclusivity ought ultimately to be retained. For instance, criterion 1 suggests that [The Department of Philosophy is located at 37°14′0″N 115°48′30″W] is *both* a social and a natural fact insofar as the corresponding statement is expressed in both social and natural vocabulary. However, criterion 2 suggests that [The Department of Philosophy is located at 37°14′0″N 115°48′30″W] is a social fact insofar as it’s social component – namely, the Department of Philosophy – is least fundamental; that is, the component that appears “last” – or “highest up” – in the relevant hierarchy. Lastly, criterion 3 suggests [The Department of Philosophy is located at 37°14′0″N 115°48′30″W] is a social fact, but now because it’s plausible that it’s about its social component; namely, the Department of Philosophy.^29^ Whichever we prefer, the availability of criteria that preserve exclusivity is sufficient for our purposes. In fact, nothing prevents us from adopting multiple criteria. Should we, we might accept that a single fact is social in one sense, and natural in another sense. Although the general model of reality’s commitment to exclusivity is controversial, then, the concerns it raises aren’t dispositive.

As a result, I’ll assume that Fig. 2 provides an adequate independent basis for answering our questions (or, minimally, that its inadequacies don’t distort them). The reader who thinks otherwise is invited to articulate an improved model.

## The social and the socially constructed

### SC to S

Let’s suppose that every socially constructed fact is like the fact that the Department of Philosophy. is on strike: socially constructed and also social. Given **Social Construction** and **Social**, **SC to S.** entails that if x depends on social goings-on, x isn’t natural. So, we can ask: Is social reality like *that*?

**Fig. 3:**
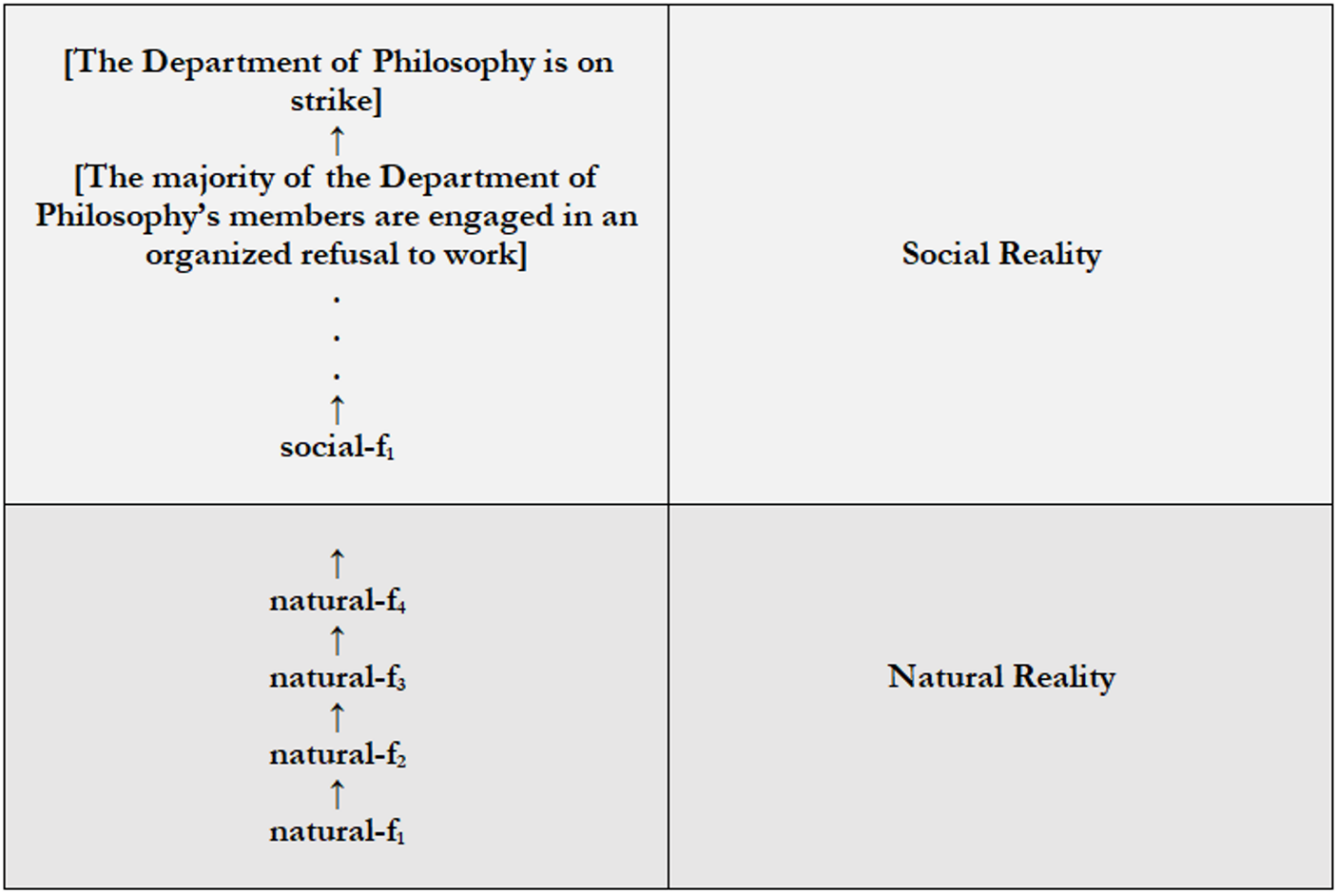
SC to S

Plausibly so. Consider Fig. 3.

Figure 3 captures the view of **§II** that [The Department of Philosophy is on strike] is a paradigmatically social and also socially constructed fact. It’s social in e.g., being about social things (e.g., the Department of Philosophy and its strike), and it depends on social goings-on; namely, on [The majority of the Department of Philosophy’s members are engaged in a coordinated refusal to work]. Because Fig. 3 is a version of the general model of reality, it follows that [The Department of Philosophy is on Strike] isn’t natural. Again, the general model implies that for any level, L, L is individuated by a particular kind, K, such that, for all x, x exists in L if and only if x is K. Thus, everything that’s social belongs to social reality, and everything that belongs to social reality is social. Clearly, [The Department of Philosophy is on strike] belongs to social reality. Thus, it can’t be natural.

The same appears to be true of [The majority of the Department of Philosophy’s members are engaged in a coordinated refusal to work]. It too is a paradigmatically social fact that’s socially constructed. Again, it appears to be about social things (e.g., a majority’s coordinated refusal to work), and it depends on social goings-on (i.e., on social-fact_1_). And because it belongs to social reality, it can’t be natural either. In fact, everything that’s true of [The Department of Philosophy is on strike] appears to be true of [The majority of the Department of Philosophy’s members are engaged in a coordinated refusal to work]. Thus, in placing facts like [The Department of Philosophy is on strike] and [The majority of the Department of Philosophy’s members are engaged in a coordinated refusal to work] in social reality, Fig. 3 implies that if x is socially constructed, x is social. What comes to the same, if x depends on social goings-on, x isn’t natural. Thus, Fig. 3 is good evidence for **SC to S**.

Insofar as we accept **Social** and **Social Construction**, that’s quite plausible. It’s difficult to see what the point of theorizing about social construction is if not to understand how *social* reality fits into an otherwise natural reality, how the *social* things are related to the natural things. But although Fig. 3 is good evidence for **SC to S**, there’s room to resist it. For it suggests a version of the controversial view of the previous section, that socially constructed facts belong to whatever level of reality to which the social goings-on on which they depend belong. And that can be resisted.

There are two kinds of cases that make trouble for **SC to S**. The first has it that there are socially constructed facts that are also natural, and the second has it that there are socially constructed facts that are also non-social. The first is a special case of the second, and the more relevant provided we accept Figs. 2 and 3. For Figs. 2 and 3 assume that there are exactly two kinds of facts – social and natural – such that a fact is non-social if and only if it’s natural. But if there are compelling cases of the second kind, not only is **SC to SC** threatened, the model of reality on which it depends is threatened. So, we should consider it separately.

As for the first kind of case, one might accept that something is socially constructed and not also social in being *natural*. An especially plausible case is one in which something that’s socially constructed is natural in being an element of a “natural social arrangement”. For instance, we can suppose that to be a wolfpack is to be a familial group consisting of individuals disposed to engage in various kinds of coordinated behavior (e.g., hunting, breeding, and rearing). Thus, the fact that a particular set of individuals – say, the Pawlsons – is a wolfpack directly depends on social goings-on; namely, their being the relevant kind of familial group. Nonetheless, one might accept that wolfpacks – and the Pawlsons – are natural phenomena. For instance, one might accept that they’re the kinds of things studied by biologists such that they’re the kinds of things we might ostend in contrast to the social.^30^ If so, **SC to S** is plausibly false: the fact that the Pawlsons are a wolfpack is a natural fact that’s also socially constructed.

As for the second kind of case, one might accept that something is socially constructed and not also social in being *non*-social. An especially plausible case is one in which something that’s socially constructed is non-social not in being an element of a “non-social social arrangement” (what could such a thing be?) but in being a non-social thing that *in*directly depends on social goings-on. Consider a naive kind of moral constructivism according to which moral principles^31^ directly depend on social goings-on. For instance, one might accept that the Principle of Utility depends on our social practices; say, in our social practices committing us to it.^32^ If so, it’s plausible that the Principle of Utility is socially constructed.

Nonetheless, the Principle of Utility has particular moral facts as consequences.^33^ For instance, it entails that it’s wrong to eat meat when eating meat produces more harm than benefit. And one might accept that the fact that it’s wrong to eat meat is a paradigmatically moral (rather than a social) fact. But insofar as it depends on the Principle of Utility, the fact that it’s wrong to eat meat indirectly depends on the social goings-on on which the Principle of Utility does (in being among its consequences). It’s therefore plausible that the fact that it’s wrong to eat meat is socially constructed, too. And because it’s a moral (rather than a social) fact, **SC to S** is false: it’s a non-social fact that’s also socially constructed.^34^

I find these kinds of cases implausible for two reasons. The first is that the offending facts – whether about wolfpacks, or wrongness – seem to be social (rather than either natural or moral) facts, and the second is that they seem not to be socially constructed. And although I think we can (and should!) resist them on both counts,^35^ the clearest case to be made against them is of the former sort: the fact that the Pawlsons are a wolfpack and the fact that it’s wrong to eat meat are social (rather than either natural or moral) facts.

Here’s the idea. As discussed in **§II**, we can disagree about what marks the social, though the kind of dependence on things we do (in the relevant sense of “do”) discussed there is a plausible candidate. Again, things can come to exist, cease to exist, and change on the basis of things we do. Again, we can create syndicates with handshakes, destroy the Salsa by censoring it, and affect a strike by agreeing to terms. Paradigmatically natural things aren’t like that. And both the fact that the Pawlsons are a wolfpack and the fact that it’s wrong to eat meat exhibit precisely this kind of dependence on things we do *ex hypothesi*.

First, it’s plausible that facts about wolfpacks are responsive to things they do. In particular, it’s plausible that if wolves have the kind of cognitive sophistication required to *coordinate* in the ways we might, wolfpacks are social. For instance, a collection of wolves might create a pack in coordinating in some relevant way, might destroy their pack in refusing to, and might change its structure to include new roles. If so, it’s plausible that the “we” in “depending on things we do” includes *them*. In other words, if wolves coordinate in the ways we do, it’s nearly irresistible to accept that wolves and their packs are as much part of the social domain as we are, and so that facts about their packs are social facts.

We can contrast wolves with creatures that don’t appear to be cognitively sophisticated enough to e.g., coordinate in the ways we might. Consider bees and the fact that a particular set of individuals – say, the Bizzmarks – is a colony. The Bizzmarks’ behavior is plausibly mechanistic; in particular, *because* it’s plausible that bees aren’t sufficiently cognitively sophisticated to coordinate in the ways we (and wolves) do. Surely, we *say* that they play roles – we call them “queens,” for instance – but it’s plausible that these are merely metaphorical uses of the relevant terms (in the way the use of “colony” to describe the way bacteria aggregate is metaphorical). But if we accept that bees and wolves differ in this respect, we’ll have grounds for including wolves and excluding bees from the social domain. And if so, it’s plausible that the fact that the Pawlsons are a wolfpack isn’t a counterexample to **SC to S**: it’s a social and also socially constructed fact.

Second, should moral conventionalism of the relevant sort be true, moral facts exhibit precisely this kind of dependence on things we do, too, albeit indirectly. That it’s wrong to eat meat depends on things we do in being consequences of the Principle of Utility, which depends on our social practices. Should our practices significantly differ, it might be that the Principle of Utility is such that it *isn’t* wrong to eat meat. If moral conventionalism is true, particular moral facts can come to exist, cease to exist, and change on the basis of things they do, too. Thus, they bear a mark of the social. It's therefore plausible that if the kind of moral conventionalism in question is true, the fact that it’s wrong to eat meat is indeed a social fact. Ordinarily, we don’t think of moral facts as socially constructed. Ordinarily, wrongness seems to have nothing to do with us. But provided the moral conventionalist says otherwise, it’s much more natural to say that although the fact that it’s wrong to eat meat *seems* like a moral fact, it’s really a social fact masquerading as a moral fact. Should we learn that moral conventionalism is true, we’ll thereby have learned that the fact that it’s wrong to eat meat is an ostensibly moral, social fact.

Of course, there are many more cases to consider. But I offer this as a challenge to those in search of others. Find a candidate natural or otherwise non-social fact that depends on social goings-on. Suppose that the way in which it depends on social goings-on is sufficient for its being socially constructed. Ask: is the fact in question *really* natural or otherwise non-social, or does it merely appear to be so? If it’s a case like either of those I’ve considered here, it merely appears to be natural or otherwise non-social. And if so, **SC to S** remains.

### S to SC

Let’s now suppose that every social fact is like the fact that the Department of Philosophy is on strike: again, social and also socially constructed. Given **Social** and **Social Construction**, **S to SC** entails that if x isn’t natural, x depends on social goings-on. So, again: Is social reality like *that*?

Plausibly not. For in combination with **Social Construction**, **S to SC** generates a regress.

Consider a socially constructed fact: [The Department of Philosophy is on strike]. It follows from **Social Construction** that [The Department of Philosophy is on strike] depends on a social fact; in this case, [The majority of the Department of Philosophy’s members are engaged in an organized refusal to work]. It therefore follows from **S to SC** that [The majority of the Department of Philosophy’s members are engaged in an organized refusal to work] is socially constructed. And, so, it follows from **Social Construction** that [The majority of the Department of Philosophy’s members are engaged in an organized refusal to work] depends on a social fact; say, [Dwayne, Dominique, and Danielle are picketing]. But, again, it follows from **S to SC** that [Dwayne, Dominique, and Danielle are picketing] is socially constructed, and, so, that *it* depends on social fact. And on and on and on.

**Fig. 4:**
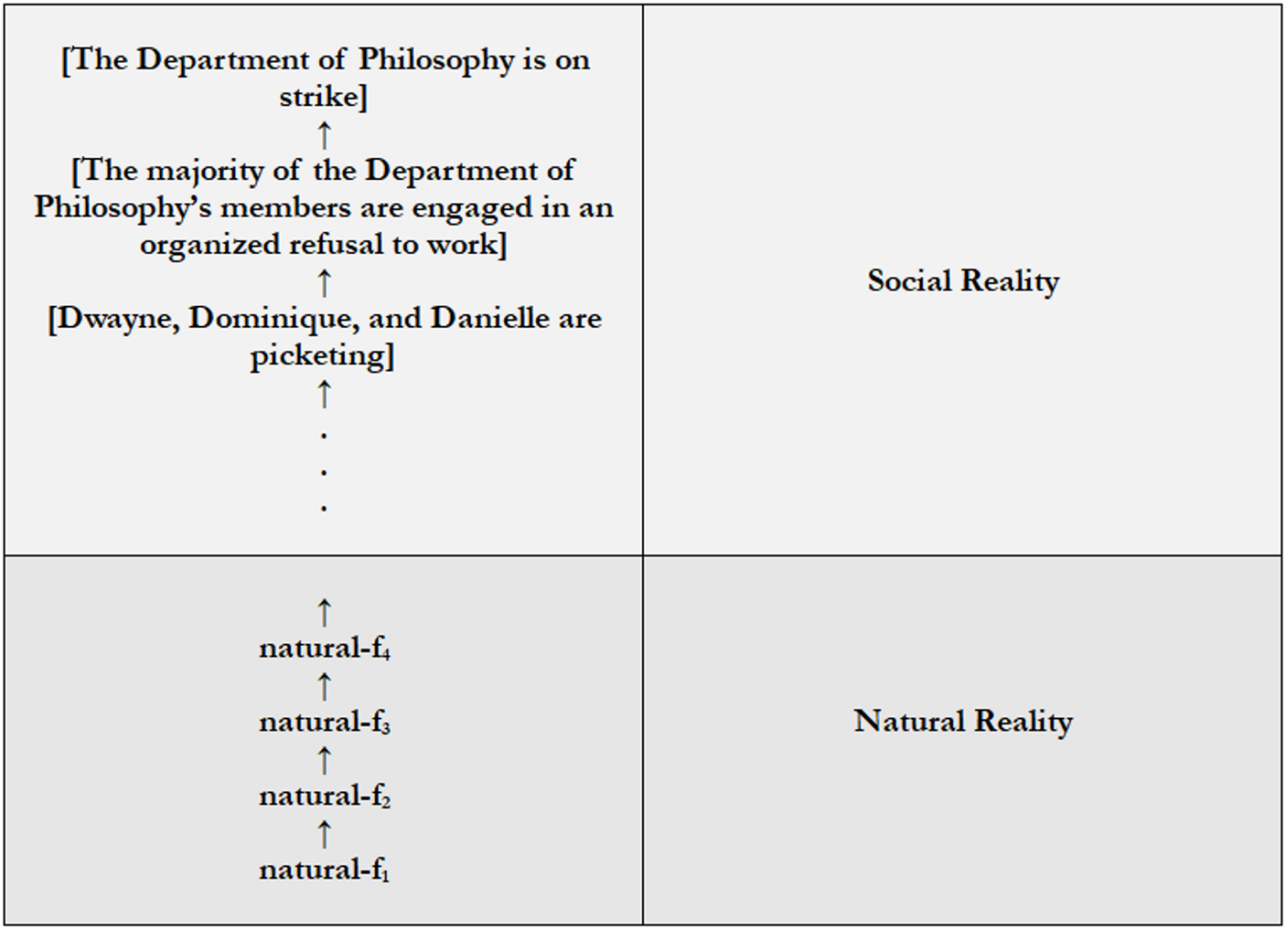
Regress

Figure 4 illustrates.

Ultimately, the regress presents us with a choice. We might countenance the regress. But insofar as we don’t, and insofar as the *combination* of **Social Construction** and **S to SC** generates the regress, we can abandon **S to SC**, **Social Construction**, or both. Here I’ll suggest we abandon **S to SC**.^36^

First, the view that social reality consists of an infinite series of social constructions in deeply counterintuitive. Part of what’s attractive about the general model of reality is that it implies that social reality and natural reality form a continuous hierarchy in which the first depends on the second. And it’s difficult to see what it would mean for social reality to depend on natural reality in the relevant sense if social reality were *bottomless*. If there were no “first” social fact, it’s difficult to understand *what* in social reality depends on natural reality, to understand *into what* natural reality is thereby hooked. It’s therefore understandable if we prefer not to countenance the regress.

Second, it’s clear that we shouldn’t abandon **Social Construction**. As suggested in **§II**, the literature on social construction is orientated around **Social Construction**. Whatever one’s account of the relevant kind of dependence, social metaphysicians agree that to be socially constructed is *in some sense* to depend on social goings-on. For instance, Haslanger (2012: Ch. 1) understands social construction in terms of a thing’s being defined by – and, so, depending for its identity – on social factors. Jonathan Schaffer (2017, 2019) and Aaron Griffith (2017, 2018) argue that for a fact to be socially constructed is for it to be grounded in social goings-on, and so for it to depend on, and to be determined and in some sense explained by social goings-on. Similarly, Asya Passinksy (*Forthcoming*) argues that for a fact to be socially constructed is for the fact that it’s grounded in whatever way it is to be *meta*-grounded in social goings-on. And Ásta 2018 understands social construction in terms of a social status’s being “conferred” on what’s thereby socially constructed, where this implies that something’s having a conferred social status depends on things the relevant conferrers do (again, in some appropriately wide sense of “do”). Dependence on social goings-on is therefore at the heart of extant accounts of social construction. Thus, if we were to abandon **Social Construction**, they couldn’t be sustained as such.

Lastly, the relatively modest view that something is socially constructed shouldn’t entail an infinite series of social constructions. For on the one hand, the fact that one fact is socially constructed shouldn’t entail that infinitely many facts are socially constructed. The fact that one fact is socially constructed should at most entail that there are two social facts: what’s socially constructed, and the social goings-on on which it depends. On the other hand, accepting that some fact is socially constructed should be compatible with the view that there’s a “first” social phenomenon, that social reality is structured in the way Fig. 3 has it. It certainly shouldn’t entail that reality is structured in the way Fig. 4 has it. Regardless of one’s attitude toward the regress itself, everyone should be entitled to the view that there aren’t infinitely many social constructions.

Here's an analogy. Suppose we accept “evolutionary” analogues of **Social Construction** and **S to SC**. On the one hand, for a phenomenon to evolve is for it to causally depend on prior biological phenomena, and, on the other hand, if a phenomenon is biological, it has evolved from prior biological phenomena. Call these **Evolution** and **B to E**, respectively. As with **Social Construction**, accepting **Evolution** doesn’t commit one to a particular account of evolution. All it commits one to is the minimal view that if one thing evolves from a second, the first causally depends on the second in the relevant way. And the combination of **Evolution** and **B to E** likewise generates a regress.

Suppose humans are an evolved species. **Evolution** entails humans causally depend on prior biological phenomena; say, on Homo Erectus. It therefore follows from **B to E** that Homo Erectus was an evolved species. But then it follows from **Evolution** that Homo Erectus causally depended on prior biological phenomena, too; on, say, Australopithecus africanus. But then it follows from **B to E** that Australopithecus africanus was an evolved species, and then, from **Evolution**, that it causally depended on further biological phenomena. And on and on and on. And again, the view that there’s one evolved species shouldn’t entail that there are infinitely many. The fact that one species is evolved should at most entail that there are two biological phenomena; namely, what has evolved (i.e., the relevant species), and the prior biological phenomenon from which it evolves (i.e., on which it causally depends). Likewise, accepting that one species has evolved should be compatible with the view that there was some first biological phenomenon from which the rest evolved. In other words, one’s account of evolution should be compatible with the view that there are finitely many evolved species, as well as that there was some initial cause of them; that is, that evolution isn’t “regressive” in the relevant way.^37^

And here’s the point. We have independent reason not to abandon either **Evolution** and **Social Construction**. Thus, if we’re disinclined to accept the biological regress, it must be because we’re disinclined to accept **B to E**. Analogously, if we’re disinclined to accept the social regress, it must be because we’re disinclined to accept **S to SC**. So, we should abandon **S to SC**.

**Fig. 5:**
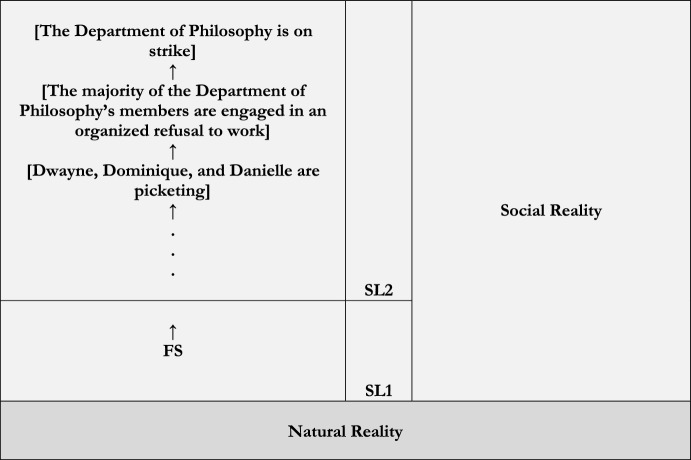
SL1 and SL2

Thus, we can regain the view that social reality doesn’t consist of an infinite series of social constructions by revising Fig. 4. Consider Fig. 5.

Figure 5 supplements Fig. 3. As before, it represents reality as consisting of the kinds of levels identified in Fig. 1, levels consisting of general kinds of facts that are hierarchically, externally structured; again, the social and the natural facts there are. Moreover, it represents reality’s levels as consisting of facts that are internally and externally structured as in Fig. 2. But now it represents each level’s internal structure as consisting of further sub-levels: here SL1 and SL2. In particular, it represents the social facts belonging to SL2 as ultimately depending on facts belonging to SL1 – whether directly or indirectly – which themselves directly depend on facts belonging to natural reality. SL2 is therefore the point at which the natural “ends” and the social “begins.” SL2 is social reality’s lower bound.^38^

And now it’s quite clear why **S to SC** is false. There’s something in social reality that *doesn’t* depend on social goings-on. Whichever facts belong to SL2 (i.e., FS) don’t depend on social goings-on. It follows from **Social** and **Social Construction**, then, that FS is social but *not* also socially constructed. For although FS is social in belonging to social reality, it directly depends on natural goings-on. Although it belongs to social reality, we can say that it’s *fundamental relative to it*. And, so, the buck stops there.

## Fundamentality relative to social reality

We should pause to appreciate the significance of this thought. If the preceding arguments are successful, the distinction between SL1 and SL2 isn’t a mere convenience. Rather, SL1 and SL2 are part of reality’s structure. And what belongs to SL1 is special. Again, it’s that in virtue of which social reality depends on natural reality. We should therefore treat it with care. Here I’ll propose an account of fundamentality relative to social reality, and I’ll use to Searle’s account to illustrate how it might be of use to social metaphysicians generally. In that way, I’ll show how the distinction between the properties of being social and of being socially constructed can be put to work.

First, we need a distinction. In particular, we need to distinguish something’s being fundamental relative to what we can call an *independent* level from something’s being fundamental relative to what we can call a *dependent* level. Suppose again that reality is as Fig. 1 has it. There reality’s physical level is independent in the sense that there’s no level on which it depends. Nonetheless, something might be fundamental relative to it. But reality’s social, psychological, biological, and chemical levels aren’t like that. These levels are dependent in the sense that there *is* some level on which they depend. As Fig. 1 has it, social reality directly depends on reality’s psychological level, which directly depends on its biological level, which directly depends on its chemical level, which directly depends on its physical level. And something might be fundamental relative to each of these levels. Whatever is fundamental relative to them is that in virtue of which they’re dependent.

We’re interested in a particular dependent level: social reality. And given Figs. 2, 3, 4, 5, social reality is contrasted with a particular *in*dependent superlevel: natural reality. As a result, we’ll want our account of fundamentality relative to social reality to reflect this distinction. I propose we begin with an account of fundamentality relative to a level generally:

**Fundamentality Relative to a Level (FL)**: for a fact, f, to be fundamental relative to a level, L, is for f to **(a)** belong to L, and **(b)** be independent of other facts belonging to L

**FL** makes no distinction between dependent and independent levels. Whatever the level, **FL** implies that something might be fundamental relative to it. We can therefore use **FL** to characterize what’s fundamental relative to a particular dependent level by specifying that on which it’s supposed to depend. For social reality, we get.

**Fundamentality Relative to Social Reality (FSR)**: For a fact, f, to be fundamental relative to social reality is for f to **(a)** belong to social reality, **(b)** be independent of other facts belonging to social reality, and **(c)** directly depend on natural goings-on^39^

Clearly, **FSR** is an instance of **FL**. But we have to add something to **FL** to ascertain what’s fundamental relative to social reality.^40^

To that end, let’s consider a particular proposal for what’s fundamental relative to social reality adapted from Searle.^41^ Searle conceives of his task as that of “assimilat[ing] social reality to our basic ontology (1995: 41)”; in his case, our basic ontology of physics, chemistry, and biology (*ibid*). He argues that “we need to show the continuous line that goes from [things like] molecules and mountains to…[things like] legislatures, money, and nation-states (1995: 41).” In a word, we need an account of how social reality fits into reality per se.

Here’s how he does it. First, he argues that there’s a precondition on the existence of social reality. As he says, the existence of social reality presupposes that some creatures (relevantly like us) have the *capacity* to “attach a sense, a symbolic function, to an object that does not have that sense intrinsically (1995: 75).” According to Searle, this is a biological capacity for collective intentionality. Second, he argues that we might deploy our capacity to attach a sense to an object that does not have that sense intrinsically by collectively agreeing to what he calls “constitutive rules.” Where X refers to an object with the relevant intrinsic features, Y to the status we attach to it, and C to the context in which we attach it, constitutive rules have the form *X counts as Y in C*. For instance, when we agree to have money, we collectively agree to treat, say, bits of metal as stores of value in a particular kind of system of exchange. Lastly, constitutive rules generate the fact that an object has the social properties it has. Money enters the ontology if and only if we agree to treat bits of metal as a store of value. Nothing is such that it’s a store of value – that it’s money – intrinsically.

Importantly, Searle’s account is iterative: some constitutive rules’ X terms refer to natural goings-on, and some to social goings-on already generated by these more fundamental constitutive rules. Thus, the constitutive rules whose X term refers to natural goings-on are special. They relate social reality to natural realty. In our terminology, *they’re* what’s fundamental relative to social reality.

First, in being constitutive rules, they’re social facts. And in being social facts, they belong to social reality. That’s **(a)**. Second, they’re independent of other constitutive rules and the social goings-on they generate. That’s **(b)**. Lastly, they directly depend on natural goings-on. That’s **(c)**. For instance, the fact that this bit of metal is money depends on the constitutive rule that counts bits of metal as stores of value in the relevant context. Because constitutive rules are themselves social facts, the fact that this bit of metal is money is a socially constructed fact. But this constitutive rule directly depends on our collectively accepting it. That is, it directly depends on natural (i.e., a psychological) goings-on. It’s thereby a social fact that directly depends on natural goings-on: it’s fundamental relative to social reality, and, so, it’s social but not also socially constructed.

Searle’s account therefore raises several interesting questions about fundamentality relative to social reality. Clearly, it provides an account of *what* might be fundamental relative to social reality (the relevant kind of constitutive rules) and why (because they’re the result of our deploying the relevant biological capacity in collectively accepting them). Of course, we might disagree. But now we see what we might be disagreeing about; namely, what’s fundamental relative to social reality.

Relatedly, Searle’s account doesn’t require that every socially constructed fact *fully* depends on some constitutive rule that’s fundamental relative to social reality. In other words, it’s compatible with his account that, for any socially constructed fact, SCF, and any constitutive rule that’s fundamental relative to social reality, FR, SCF merely partly depends on FR. SCF might depend on exactly one thing. Nonetheless, his account reveals that every socially constructed fact depends – whether fully or partly – on some constitutive rule that’s fundamental relative to social reality. And this is reflected in **FSR**. As far as **FSR** says, what’s fundamental relative to social reality might generate socially constructed facts in either of these ways as long as it’s independent of them. As a result, **FSR** accommodates disagreements about possible “routes” from what’s socially constructed to what’s fundamental relative to social reality whether or not its constitutive rules that are fundamental relative to social reality.

We can put this a bit more precisely and a bit more neutrally. On the one hand, it might be that socially constructed facts are related to what’s fundamental relative to social reality in the following way:

**Strong Socially Constructed**: For a fact, SCF, to be socially constructed is for SCF to belong to social reality’s second level (SL2), and for SCF to fully depend on some fact that’s fundamental relative to social reality

Roughly, **Strong Socially Constructed** says that everything that lives in SL2 ultimately – that is, fully, although indirectly – depends on something that lives in SL1. In this case, what’s fundamental relative to social reality is “complete” relative to social reality.^42^

On the other hand, however, it might be that socially constructed facts are related to what’s fundamental relative to social reality in the following way:

**Weak Socially Constructed:** For a fact, SCF, to be socially constructed is for SCF to belong to social reality’s second level (SL2), and for SCF to partly depend on some fact that’s fundamental relative to social reality

In this case, although what’s fundamental relative to social reality *isn’t* complete, it’s part of that on which every fact in SL2 depends.

Lastly, Searle’s account raises interesting questions about the natural goings-on on which what’s fundamental relative to social reality directly depend. For Searle, the relevant natural (i.e., biological) capacity to attach a sense to an object that does not have that sense intrinsically is presupposed by the existence of social reality. There would be no social reality if there weren’t creatures with this capacity. However, the natural goings-on on which the relevant “fundamental” constitutive rules directly depend consist of the *deployment* of that capacity in our collectively accepting them. For Searle, these are psychological goings-on, consisting of collections of individuals having individual intentions with, as it were, collective content (i.e., I intend that we intend, you intend that we intend, etc.). But again, we can disagree. And again, now see what we might be disagreeing about; namely, the natural goings-on on which what’s fundamental relative to social reality directly depend.

Importantly, this aspect of Searle’s view makes him an “individualist” rather than a “collectivist.” To that extent, **FRS** clarifies their longstanding disagreement.^43^ Roughly, individualists claim that social reality’s “determination base” consists of facts about individual agents (e.g., facts about their psychologies) and facts about individual agents only.^44^ Collectivists claim that its determination base consists of *more* than facts about individual agents; it might consist of facts about groups, structures, and/or resources, too (Haslanger, 2012; Epstein 2016). And **FRS** reveals that this might mean several things.

On the one hand, there’s a meaningful sense in which the natural goings-on on which what’s fundamental relative to social reality directly depend comprise its determination base. That’s the sense in which what’s fundamental relative to social reality – say, constitutive rules – directly depend on their collective acceptance. On the other hand, however, there’s a meaningful sense in which what’s fundamental relative to social reality comprises its determination base. That’s the sense in which what’s *socially constructed* ultimately depends on, say, the relevant constitutive rules. But if we fail to draw the distinction between the properties of being social and of being socially constructed this will remain ambiguous, and in a way that matters.

The view on which the relevant natural goings-on consist of facts about individual agents is quite unlike the view that what’s fundamental relative to social reality does. Because the former are *natural*, they have to be particular kinds of facts; for instance, facts about the relevant agents’ psychologies. But because what’s fundamental relative to social reality is *social*, the facts in question have to be of other kinds; for instance, facts about what counts as what in which context (i.e., constitutive rules). And, so, **FRS** reveals that the individualism/collectivism debate has two faces.

For individualism with respect to the relevant natural goings-on doesn’t entail individualism with respect to what’s fundamental relative to social reality, or conversely. We can as meaningfully disagree about how social reality depends on natural reality as we can about how reality’s psychological level depends on its biological level. It’s plausible that *these* are disagreements about the *nature* of the social and biological properties involved in the facts that belong to each of these levels. It might be that SL1 consists of facts about individual agents. But that alone says nothing about *what* they depend on. For instance, it might be in the nature of the social properties that figure in SL1 – say, constitutive rules – that these facts depend on facts about either the relevant individuals’ psychologies, or about more than that; perhaps about the broader communities in which they’re embedded. Likewise, it might be that SL1 consists of facts about more than constitutive rules; say, in facts about groups, structures, and/or resources. For it might be in the nature of the properties that figure in SL1 that these facts depend on facts about either the relevant individuals’ psychologies or about more than that. Thus, it’s important to emphasize that these distinctions are visible to precisely the extent that we distinguish SL2 from SL1 from the natural goings-on on which SL1 depends.

In sum, **FSR** permits disagreements about *what’s* fundamental to social reality. Whatever it is, it must be such that socially constructed facts ultimately depend on it, as well as that it directly depends on natural goings-on. Searle’s account is important in suggesting one kind of thing that might play this role. Additionally – and relatedly – **FSR** allows that socially constructed facts might depend on what’s fundamental relative to social reality in different ways; in particular, by ultimately, fully depending on them (per **Strong Socially Constructed**), or by being ultimately, partly depending on them (per **Weak Socially Constructed**). Searle’s account is telling in being compatible with each. Lastly, **FSR** interacts directly with a longstanding debate between individualists and collectivists. In particular, it reveals an ambiguity in talk of social reality’s determination base and, so, gives the debate a distinctive shape. And once we see one of these sources of disagreement, we’re likely to see many more. Ultimately, my point isn’t to settle the questions they raise. Rather, my point is that **FSR** permits meaningful disagreements about social reality’s structure that aren’t quite visible until we draw the distinction between the properties of being social and of being socially constructed. So, I say, let’s draw it.

## Conclusion

In this paper, I’ve both articulated a model of social reality, and used it to argue that although everything that’s socially constructed is also social, not everything that’s social is also socially constructed. Some social things aren’t socially constructed. Some are fundamental relative to social reality. I’ve then articulated an account of fundamentality relative to social reality, and used Searle’s account as an illustration of how it works. Ultimately, I hope to have shown that by distinguishing the property of being social from the property of being socially constructed, we’ll have availed ourselves of a distinction that is of general use to social metaphysicians.

## Footnotes

1 I use "goings-on" neutrally to refer to things of various kinds (e.g., objects, properties, facts, states of affairs, and so on).

2 It’s important to note that both Payton 2022’s argument for a sufficient condition on the property of being social, and Passinksy *Forthcoming*’s argument for an account of social construction presupposes a distinction between the properties of being social and of being socially constructed. But I’m interested in the distinction itself.

3 The *term* “social construction” isn’t as recent. But as Hacking 1999 emphasizes, “the point” of theorizing about social construction had long been to raise consciousness about the evitability of allegedly inevitable (read: natural) phenomena (e.g., race and gender). But that’s not what I’m interested in. Rather, I’m interested in social reality, and I follow the more recent trend of using the term “social construction” to refer to the kind of dependence that captures its structure. More on this shortly.

4 It’s tempting to insist that collective intentions are social in being *collective*. But it’s unclear whether something’s being collective in the relevant sense entails that its social. For instance, the property of being scattered is in some sense collective; say, in the sense that only collections of things can be scattered. But the property of being scattered isn’t social, and the relevant collections of things needn’t be either. Thus, more needs to be said about whether collection intentions are social. In fact, that’s part of my point.

5 The simplest cases are of recessions and racism. Roughly, the idea is that we don’t have to intend that an economy be in a recession for it to be in a recession, and we don’t have to intend that our society be racist for it to be racist. However, it’s important to note that Khalidi’s distinction is finer-grained than Thomasson’s. In particular, he argues that, like social byproducts, Type 1 social kinds are such that *no one* has to have *any* of the relevant kinds of attitudes (e.g., collective intentions) toward them in order for them to exist. But Type 2 social kinds are such that *someone* has to have the relevant kinds of attitudes toward them in order for them to exist, though not toward their instances in order for *them* to exist.

6 The unclarity is compounded by the fact that elsewhere Haslanger accepts that to be socially constructed is in some sense to be *defined* by social goings-on (2012: Ch. 1).

7 Payton 2024 rightly points out that it can’t be that to be social *is* to be socially constructed, or that to be socially constructed *is* to be social. As she says, if being socially constructed has anything to do with being social, “it needs to be the case that being *social* is one thing, and being *socially constructed* is another (2024: 742).”

8 When we focus on Haslanger’s definitional account of social construction (see footnote 6), that’s implausible. Presumably, what it is to have a particular race doesn’t depend on us in *this* way.

9 Here I use the term “natural” non-technically. Many use “natural” to refer to a structural feature of reality; in particular, to a property had in degrees by that which “carves at the joints”. But I use it to refer to a domain consisting of things that are relevantly non-social; to the kinds of things e.g., a physicist or a chemist or a biologist might study. This closely resembles the sense of “natural” in play in metaethics. See Shafer-Landau 2003, Smith 1994, and Copp 2007 for representative examples.

10 An interesting bit of trickiness: We can suppose there are mathematical things; for instance, that there are numbers. Intuitively, numbers are both non-natural and non-social. Thus, if non-natural things are social, numbers are social. But we’ve supposed that numbers are *non*-social. And if numbers are non-social, they’re natural. But, again, they’re non-natural! This implies that our contrast isn’t merely exclusive. It’s exhaustive, too. More on this in **§III**.

11 It’s especially common to claim that whereas the social things are in some sense the evitable things, the natural things are in some sense the inevitable things (cf. Antony 1998; de Beauvoir 1949; Diaz-Leon 2015; Hacking 1999; Haslanger 2012). But that won’t do. Being inevitable is something like being necessary when we hold the laws of nature fixed. Thus, on the present proposal, everything that’s necessary when we hold the laws of nature fixed is natural. If so, the truth of determinism would entail that nothing is social. For if everything were inevitable (per determinism), and if everything that’s inevitable is natural (per the present proposal), the truth of determinism would entail that everything is natural, and so that nothing is social. Of course, it’s possible that nothing is social. But it’s hard to accept that *determinism* is what would settle it.

12 Footnote 9 aside, there are many alternatives. For instance, we might say that (1) whereas social things are subject to “looping effects” (Hacking 1999; Khalidi 2010), natural things aren’t. We might say that (2) whereas social things are what’s studied by what we recognize as “the social sciences,” natural things are what’s studied by what we recognize as “the natural sciences”. And we might say that (3) whereas social things are essentially collective (Gilbert 1989), the natural things aren’t. None of these is without its problems (see footnote 3 for a particular concern with (3)), but we shouldn’t expect otherwise.

13 Variants of this thought are common (cf. Searle 1995, 2010; Tuomela 2002; Thomasson 2003; Khalidi 2010, 2013; Ásta 2018; Passinsky 2020). As Searle suggests, social goings-on are the kinds of things that exist “because we believe them to exist (Searle 1995: 1),” though we can substitute “believe” for other attitudes that are involved in things we do generally.

14 I break with convention and use “non-causal” to refer to so-called “constitutive” social construction. Ordinarily, metaphysicians use “constitution” to refer to the relation between a thing and its parts; for instance, to the relation between a statue and a lump of clay. But the relation between e.g., the fact that the Department of Philosophy is on strike and the fact that the majority of its members are engaged in a coordinated refusal to work isn’t *that* relation. So, we’ll want a different term to refer to it. “Non-causal” is mine since “non-causal” encompasses a wider class of relations – *including* constitution – in terms of which accounts of social construction might be cast.

15 I relevant use of “are” and “in virtue of” are intended to be neutral expressions of non-causal dependence. But metaphysicians are likely to read them as expressions of quite particular kinds of non-causal dependence; namely, as definition and grounding, respectively. And although there are definitional (Haslanger 2012) and grounding (Schaffer 2017, 2019; Griffith 2017, 2018; Passinsky *Forthcoming*) accounts of non-causal social construction in the literature, I don’t mean to suggest that either is correct. I only use these expressions to point readers in their general direction.

16 For instance, Hacking 1999; Haslanger 2012; Schaffer 2017, 2019; Griffith 2017, 2018; Asta 2018; Passinsky *Forthcoming*. However, it’s worth pointing out that Passinsky *Forthcoming*’s account of social construction indirectly conforms to **Social Construction**. According to Passinsky, roughly, for x to be socially constructed is for social goings-on to figure among x’s meta-grounds. Thus, it’s consistent with her account that x *directly* depends on natural goings-on in being fully, immediately grounded in them. Nonetheless, her account requires that all socially constructed facts indirectly depend on social goings-on in being meta-grounded in them in the relevant way. Thanks to an anonymous referee for pressing me on this.

17 Note that **Social Construction** provides a necessary condition on accounts of the property of being socially constructed. And that’s intentional. Social metaphysicians *shouldn’t* accept that depending on social goings-on is sufficient for being socially constructed. It’s now common to accept that things like sets containing social goings-on (e.g., {Supreme Court}) depend on precisely those social goings-on (i.e., the Supreme Court). And it’s difficult to accept that a set could be socially constructed. See Schaffer 2017, Passinsky 2020, *Forthcoming*, and Pagano 2021, *Forthcoming* for argument.

18 Because the general model of reality is that according to which reality consists of a hierarchy of levels, what counts as a *version* of it depends on how one conceives of levels (say, what they are and which they are), and how one conceives of the relevant hierarchy (again, what it is and which it is). Oppenheim and Putnam’s version of the model is therefore as optional as mine.

19 I appeal to a version of this general model of reality in my *Forthcoming* as well, though I don’t develop it further there.

20 Figure 1 represents reality’s levels as if the converse it’s true, too; namely, that if one level is “above” a second, the first either directly or indirectly depends on the second. But that’s controversial. Jaegwon Kim 2002 argues that, contrary to Fig. 1, some of reality’s levels are entirely independent of others; for instance, that reality’s social level is entirely independent of e.g., its geological level. Of course, Fig. 1 doesn’t contain geological goings-on. But insofar as it could and/or should, Kim’s concern recommends we adopt a “branching” hierarchy – in which one level can be entirely independent of a second – rather than the kind of “layercake” hierarchy represented in Fig. 1. Ultimately, the version of the general model of reality *I* accept isn’t committed to the view that reality’s levels form a layercake in a way that raises *this* version of Kim’s concern. But see **§IV.i.**. for discussion.

21 For instance, it’s natural to understand ‘↑’ as *grounding* such that reality’s structure has whatever logical properties *it* has. And in fact, social metaphysicians have grown increasingly fond of using grounding to understand the metaphysics of social reality. See e.g., Epstein 2016; Schaffer 2017, 2019; Griffith 2017, 2018; Passinsky 2020, *Forthcoming*; Pagano *Forthcoming*.

22 See Pagano (ms) for discussion the claim that “the social depends on the natural, but not conversely”.

23 I’ve represented ↑ as holding between single facts, but that’s dispensable. For instance, if ↑ is grounding, it’s standard to accept that it holds between a single fact – namely, what’s grounded – and a plurality of facts – whether singular or non-singular – that grounds it.

24 Fabrice Correia characterizes a relation between kinds of facts he calls *foundation*. Roughly, for a kind of fact, K_2_, to be founded in a kind of fact, K_1_, is for every K_2_ to be grounded in some K_1_, for no member of K_1_ to be grounded in K_2_, and for K_1_ and K_2_ to be non-overlapping (Correia 2021: 10). Insofar as we think of levels as sets of kinds of facts, I’m happy to countenance the notion of foundation.

25 See Kim 2002, Thomasson 2014, and Potochnik 2021 for representative examples.

26 Because the general model isn’t committed to the existence of any particular set of levels (i.e., because we can add and subtract levels as we see fit), exhaustivity is significantly less controversial.

27 Thanks to Kit Fine for this suggestion.

28 Thanks to Jonathan Schaffer for this suggestion.

29 Ultimately, this is the criterion I prefer. But there’s still a problem. For it’s unclear why a mixed fact couldn’t have a mixed *subject matter*. For instance, [The Department of Philosophy and the location referred to by” 37°14′0″N 115°48′30″W” exist] seems to be about both the Department of Philosophy *and* the relevant location. If so, insisting on the aboutness criterion is unhelpful. I suspect there are ways to accommodate such cases. For instance, it’s possible to insist that this fact is directly about “unopinionated” existence and, so, merely indirectly about what happens to exist (i.e., the Department of Philosophy and the relevant location, respectively). But that’s a can of worms if anything is.

30 This case is adapted from one Passinsky *Forthcoming* considers. She accepts that facts about wolfpacks are *both* social and natural, and denies that they’re socially constructed. Payton 2022 makes similar remarks.

31 By “principle” I mean something that encodes necessary and sufficient conditions on e.g., being right. I don’t mean something like a rule of thumb.

32 The general kind of view I have in mind often goes by the name of moral *conventionalism*, and is commonly associated with Harman 1996, Oddie 1999, Verbeek 2008. There are of course moral *constructivists*, but they tend not to accept that moral facts depend on *social* goings-on. (See Bagnoli 2021 for an excellent survey of the history of moral constructivism.) Moral conventionalism is therefore the closest thing we have to the kind of view that makes trouble for **SC to S**.

33 There’s a general question about whether and how moral facts depend on moral principles. For instance, it’s controversial whether particular moral facts depend on the Principle of Utility in the way that e.g., facts about strikes depend on facts about coordinated refusals to work. As a result, I speak of the Principle’s *consequences* in order to remain neutral on the question of dependence. I assume only that if particular moral facts depend on the Principle of Utility, they’re at least among its consequence. See Berker 2019 for discussion.

34 This case is deeply inspired by Raven 2024’s discussion of social modality. Note however that Raven discusses *logical* conventionalism rather than moral conventionalism. But my response to logical conventionalism is the same.

35 The trouble with insisting that they aren’t socially constructed is that it seems to me we shouldn’t accept that facts about wolfpacks or about what’s right and wrong can’t be socially constructed. Although I’ll argue that my imagined interlocutors’ cases are bad counterexamples to **SC to S**, they’re bad for being implausible, not for being unintelligible.

36 Schaffer 2019 notices a similar regress that’s supposed to threaten Brian Epstein 2016’s grounding/anchoring account of how social facts arise. However, it’s important to emphasize that the regress he notices is unlike the much more general regress represented in Fig. 4. On the one hand, it’s unclear whether Epstein’s account is intended to be an account of *social construction*. He certainly doesn’t use the term “social construction” to talk about how he thinks social facts arise when he proposes it, and he later (2024: §2.2) suggests that the “anchoring” end of his grounding/anchoring account is sufficient for social construction. Thus, it’s unclear whether Schaffer’s regress *is* a regress of social constructions. On the other hand, the solution to the regress that Schaffer suggests is to adopt a framework that’s compatible with multiple models of social construction, including those that allow that some social facts are (presumably, fully immediately) grounded in natural goings-on. But this doesn’t itself imply a distinction between the properties of being social and of being socially constructed.

37 Thanks to Jonathan Schaffer for discussion.

38 It’s possible that social reality has more than two sub-levels. For instance, it’s possible that Thomasson’s products belong to SL2 and her byproducts to an additional SL*3*. But that’s compatible with the model so long as it retains its lower bound in SL1.

39 It’s important to note that **FRS** isn’t the view that what’s fundamental relative to social reality is whatever social facts belong to social reality are least fundamental. For it’s plausible that one social fact might be more fundamental per se than another while not being *more* fundamental relative to social reality. See Pagano (ms) for argument.

40 Because we’re assuming that natural reality is independent, we don’t have to add anything to **FL** to get what’s fundamental relative to *it*. For whatever is fundamental relative to an independent level is simply (or: absolutely) fundamental.

41 Of course, Searle’s isn’t the only account from which we can extract a proposal for what’s fundamental relative to social reality. For instance, it’s plausible that proponents of what has come to be called the “constraints and enablements” framework (Ásta 2018; Jenkins 2023) accept that some constraints and enablements – however they’re conceived – are fundamental relative to social reality because some of them directly depend on the attitudes of some of society’s members (e.g., a particular individual-with-standing’s speech on the appropriate occasion). Because the details of this framework are complex, I’ll leave it to those who find the framework I’m proposing useful to sort them out.

42 The notion of a complete set of facts is technical. Roughly, a complete set of facts is that of a set of facts on which everything depends, *not* of a set of facts that’s independent. A complete set of facts “provides a blueprint for reality (Schaffer 2010: 39)” in the sense that their obtaining is sufficient for everything else to obtain. Completeness is arguably a feature of what’s absolutely fundamental (though it’s as arguable that it isn’t *what it is to be* absolutely fundamental; see Bennett 2016: Ch. 5 for argument). **Strong Socially Constructed** therefore relativizes completeness to a level. It says that duplicating the facts that live in SL1 suffices to duplicate all else in social reality; namely, the facts that live in SL2.

43 See Epstein 2024 for an excellent overview of this longstanding – and many-faceted! – debate.

44 I’m intentionally eliding a distinction between so-called “ontological” and “methodological” holism and individualism. Ordinarily, this distinction is drawn in terms of whether anything other than individuals exists, on the one hand, and whether there are genuinely social explanations, on the other hand. Given the systematic relations between the kind of dependence I’m interested in and explanation, however, there’s far less daylight between the two than previously thought.
